# Predictors of recurrence after open excision of wrist ganglion cysts: an MRI-based and clinical analysis

**DOI:** 10.1186/s12893-026-03562-3

**Published:** 2026-02-05

**Authors:** Salih Kaya, Gürkan İden, Recep Taşkın, Mehmet Ali Dursun, Basri Pür, Bilal Karabak, Uğur Kayık

**Affiliations:** 1 Department of Orthopedics and Traumatology, Erzurum City Hospital, Erzurum, Turkey; 2 Department of Orthopedics and Traumatology, Iğdır State Hospital, Iğdır, Turkey; 3 Department of Orthopedics and Traumatology, Kastamonu University, Kastamonu, Turkey; 4 Department of Orthopedics and Traumatology, Tavşanlı State Hospital, Kütahya, Turkey

**Keywords:** Wrist ganglion cyst, Open surgical treatment, Recurrence predictors, Magnetic resonance imaging

## Abstract

**Background:**

Wrist ganglion cysts (GCs) are the most common soft-tissue masses of the hand and wrist. Although surgical excision is considered the most effective treatment, recurrence remains a clinical concern. Anatomical and demographic predictors of postoperative recurrence are not well established.

**Methods:**

We included 347 patients who underwent surgical excision of wrist GCs between 2015 and 2023, with a minimum follow-up of 24 months. Clinical data (age, sex, side, hand dominance) and magnetic resonance imaging (MRI)–based topographic features (volume, surface area, wall thickness, location, longest and shortest diameters, aspect ratio, and distance to the adjacent joint) were recorded retrospectively. The primary outcome was cyst recurrence at a minimum follow-up of 24 months, defined as clinically or radiologically confirmed return of the cyst. Statistical analyses included chi-square tests, t-tests or Mann–Whitney U tests, and univariable and multivariable logistic regression analyses. All analyses were performed in the Python (Google Colab) environment using the pandas, NumPy, SciPy, and statsmodels libraries.

**Results:**

The overall recurrence rate at a minimum follow-up of 24 months was 8.6% (30 of 347). Dominant-hand involvement was significantly associated with recurrence (χ² *p* = 0.006). In age-adjusted logistic regression, dominant-hand involvement increased the risk (OR = 6.51; 95% CI, 1.87–22.63; *p* = 0.003). Cyst distance to the adjacent joint was significantly shorter in recurrent cases (mean 7.6 mm vs. 8.1 mm; t-test *p* = 0.001, Mann–Whitney U *p* = 0.021). Based on age-adjusted logistic regression, each additional millimeter of cyst-to-joint distance conferred a 41% relative reduction in recurrence risk (OR = 0.59; 95% CI, 0.39–0.90; *p* = 0.013).

**Conclusions:**

MRI-based evaluation of cyst-to-joint distance and consideration of dominant-hand involvement may help identify patients at increased risk of recurrence after surgical excision of wrist GCs. Incorporating these factors into preoperative planning may optimize surgical strategy, guide follow-up, and improve patient counseling.

**Supplementary Information:**

The online version contains supplementary material available at 10.1186/s12893-026-03562-3.

## Introduction

Ganglion cysts (GCs) are the most common soft-tissue masses of the hand and wrist and typically present as palpable swellings that may cause pain, tenderness, reduced grip strength, or paresthesia due to local nerve compression [1–3]. Although some lesions remain asymptomatic, persistent discomfort, functional limitation, and cosmetic concerns often prompt medical evaluation. Nonoperative management—including observation, splinting, aspiration, and steroid injection—is frequently attempted but is associated with high recurrence rates, reported in some series to exceed 27–56% [4–6]. Consequently, surgical excision is generally considered the most definitive treatment, offering the lowest recurrence rates compared with conservative options [7–9].

Several anatomical and demographic factors have been proposed to influence recurrence risk, such as cyst size, location, and relationship to adjacent joint structures, as well as patient age, sex, and hand dominance [9, 10]. Nonetheless, the current evidence remains limited and inconsistent. As a result, identifying reliable predictors of postoperative recurrence is essential to improve preoperative risk assessment and patient counseling. However, comprehensive studies integrating detailed magnetic resonance imaging (MRI)-based topography with clinical factors to predict recurrence are lacking.

The present study aimed to evaluate the recurrence rate at a minimum follow-up of 24 months of surgically treated wrist GCs and to analyze the association of recurrence with demographic characteristics and MRI–based topographic features of GCs.

## Methods

### Study population and patient selection

**Fig. 1:**
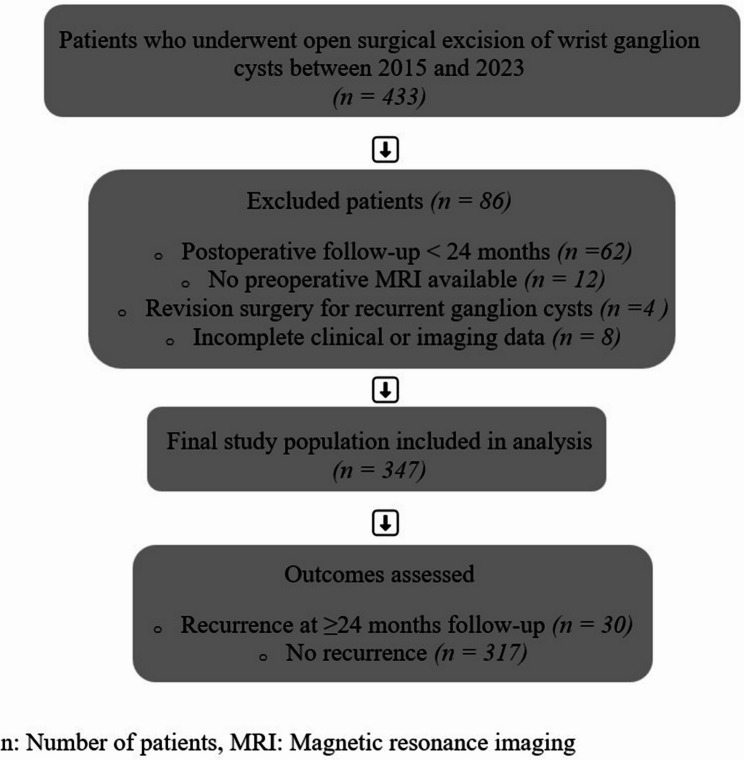
Patient selection flow diagram

We retrospectively screened 433 patients who underwent surgical excision of wrist GCs at a single tertiary referral center between 2015 and 2023. To ensure a minimum follow-up of 24 months, only patients who completed at least 24 months of follow-up were included. After applying the predefined inclusion and exclusion criteria, 347 patients were included in the final analysis. Exclusion criteria comprised revision surgery for recurrent GCs, lack of preoperative MRI, follow-up duration of less than 24 months, and incomplete clinical or imaging data (Fig. 1).

### Data collection

Clinical variables (age, sex, side, and hand dominance) and MRI-based topographic measurements (cyst volume, surface area, wall thickness, anatomical location, shortest and longest diameters, aspect ratio, and cyst-to-adjacent joint distance, measured in millimeters) were extracted from preoperative imaging and operative records.

### MRI-Based measurements and image analysis

**Fig. 2:**
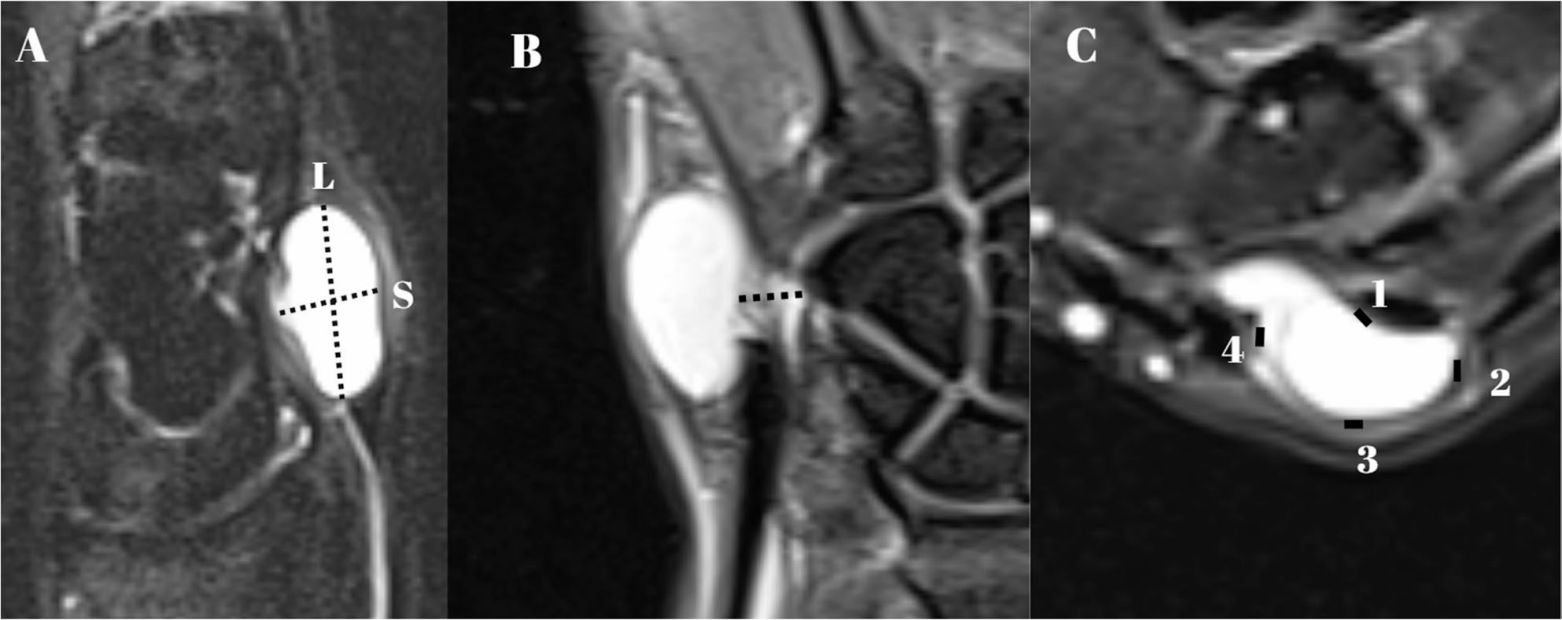
Composite MRI T2 images demonstrating topographic measurements of the ganglion cyst. (A) The dashed line labeled L denotes the longest diameter of the cyst; the dashed line labeled S denotes the shortest diameter (B) The minimal perpendicular distance from the outer cyst wall to the radiocarpal joint capsule (C) Cyst wall thickness measured at four quadrants, labeled 1, 2, 3, and 4. All panels are derived from T2-weighted wrist MRI; imaging planes are indicated on the panels, and scale bars are provided L, longest diameter; S, shortest diameter

Preoperative wrist MRI examinations were reviewed on a calibrated PACS viewer using DICOM pixel spacing. All measurements were performed using in-plane distances derived from DICOM pixel spacing to ensure consistency across routine clinical MRI examinations acquired over the study period. Axial and sagittal T2 fat suppressed images with a slice thickness of 3 mm were used for all measurements. For each cyst, the slice with the largest cross-sectional area was selected. The longest diameter (L) and the shortest diameter (S) were measured on that slice, and the aspect ratio was calculated as L divided by S. The cyst to joint distance was defined as the minimal perpendicular distance from the outer cyst wall to the nearest point on the adjacent joint capsule or cartilage, with measurements consistently performed in-plane to minimize partial volume effects. The adjacent joint was defined as the nearest articular surface to the cyst on MRI, irrespective of the presumed joint of origin. Given the retrospective design and variability in cyst morphology, the precise joint of origin and stalk attachment could not be reliably identified in all cases. Wall thickness was measured at four quadrants on the same slice and summarized as minimum and mean values. When the stalk was visible, stalk length was measured from the capsular origin to the cyst wall, and the capsular origin location was recorded as dorsal or volar. Volumetric measures were obtained by semi-automated segmentation on contiguous slices with manual correction, from which volume and surface area were computed with consistent window and level settings. All measurements were performed independently by two fellowship-trained orthopaedic surgeons who were blinded to recurrence status and other outcomes; values were averaged, and interobserver reliability was quantified using intraclass correlation coefficients (ICCs) with 95% confidence intervals, based on a two-way random-effects model with absolute agreement. This approach was used to support measurement reproducibility, particularly for small absolute distance differences. Scale bars were displayed on stored images, and all distances are reported in millimeters (Fig. 2).

### Surgical technique

**Fig. 3:**
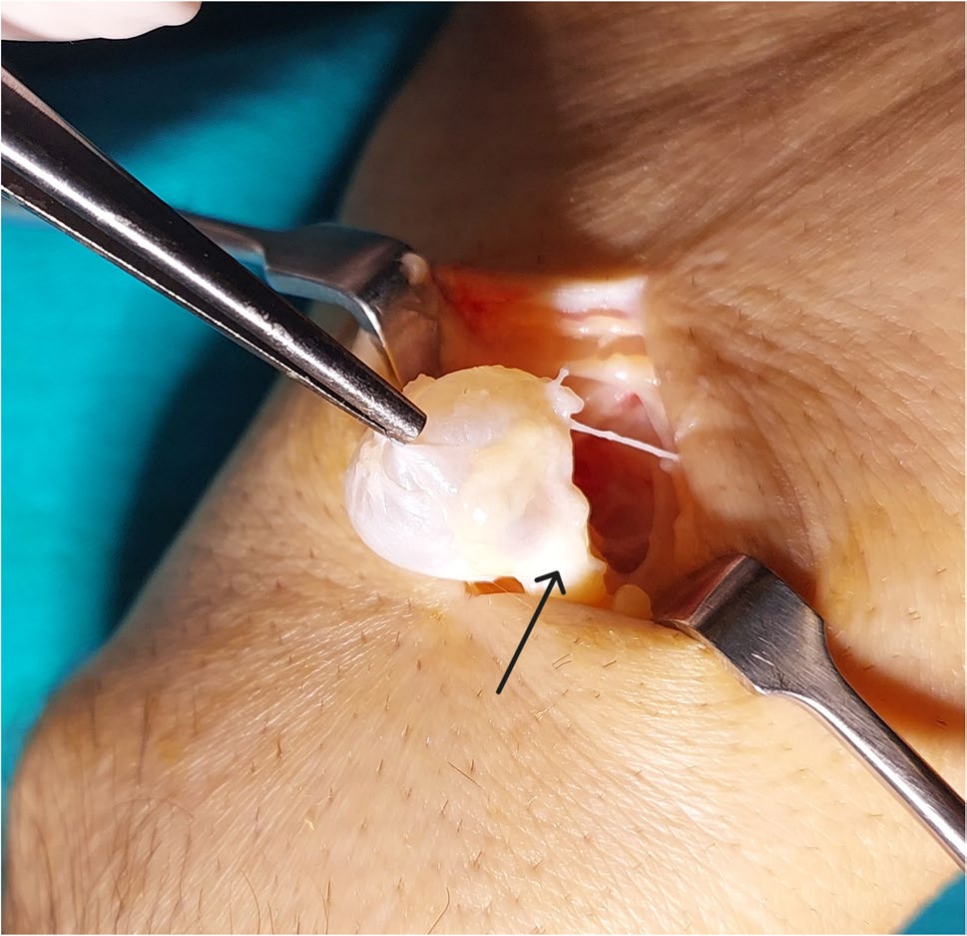
Intraoperative photograph demonstrating the cyst stalk at its capsular origin (black arrow) before excision. The stalk and a small cuff of capsule were removed en bloc.

All cases were treated with open excision. The approach (dorsal or volar) was selected according to cyst location. After tourniquet inflation and a skin incision centered over the cyst, careful dissection was performed to identify the cyst stalk and its capsular origin (Fig. 3). The cyst, its stalk, and a small cuff of joint capsule were excised en bloc; the capsular origin was cauterized or curetted at the surgeon’s discretion. The wound was irrigated and closed in layers. Postoperatively, the wrist was immobilized using a splint or compressive bandage for 1–2 weeks, followed by early range-of-motion exercises.

### Outcome definition

The primary outcome was cyst recurrence at a minimum follow-up of 24 months postoperatively, defined as the reappearance of a clinically palpable ganglion at the operated site, with or without radiologic confirmation on MRI, as documented in outpatient records.

### Statistical analysis

Statistical analysis was performed in a Google Colab environment using Python libraries including *pandas* (data preprocessing), *numpy* (numerical computations), *scipy.stats* (normality tests, t-tests, Mann–Whitney U, chi-square), and *statsmodels* (univariate and multivariate logistic regression). Categorical variables were compared using chi-square tests, and continuous variables were analyzed with t-tests or Mann–Whitney U tests after Shapiro–Wilk normality checks. Given the limited number of recurrence events, multivariable logistic regression was considered exploratory. Variables were selected a priori based on clinical relevance rather than solely on univariable significance. Age was prespecified as the primary a priori confounder and included in the primary adjusted model. Sex and cyst location were evaluated in separate sensitivity analyses due to events-per-variable (EPV) constraints. To reduce the risk of overfitting, the number of predictors in the primary model was restricted to maintain an EPV ratio of approximately 10.

### Ethics statement

The study was conducted in accordance with the Declaration of Helsinki. Ethical approval was obtained from the institutional review board (IRB approval no: 2025/08–221/date: 10.07.2025), and written informed consent for participation and data use was obtained from all patients prior to inclusion.

## Results

A total of 347 patients (mean age ± SD: 42.5 ± 16.3 years; range: 15–69) who underwent surgical excision of wrist GCs were included. Of these, 240 (69.2%) were female and 107 (30.8%) male. The right wrist was affected in 180 cases (51.9%) and the left in 167 (48.1%). The dominant hand was involved in 228 patients (65.7%). Most cysts were located on the dorsal aspect (67.7%), while 32.3% were volar. The median cyst volume was 2.61 ml (IQR 1.58–3.96), median surface area 284 mm² (IQR 201.9–386.7), and median wall thickness 1.81 mm (IQR 1.07–2.45). The median longest diameter was 24.9 mm (IQR 17.2–34.6), the median shortest diameter was 14.1 mm (IQR 9.6–20.3), and the median aspect ratio was 1.74 (IQR 0.97–2.76) (Table 1).

**Table 1:** Demographics and MRI-based topographic characteristics

Variable	Overall
Patients, n	347
Recurrence at minimum 24 months, n (%)	30 (8.6%)
Follow-up months, median [IQR]	53 [40.5–68]
Age, mean ± SD (min to max)	42.5 ± 16.3 (15 to 69)
Female, n (%)	240 (69.2%)
Male, n (%)	107 (30.8%)
Right side, n (%)	180 (51.9%)
Left side, n (%)	167 (48.1%)
Dominant hand involved, n (%)	228 (65.7%)
Dorsal location, n (%)	235 (67.7%)
Volar location, n (%)	112 (32.3%)
Longest diameter (mm), median [IQR]	24.9 [17.2–34.6]
Shortest diameter (mm), median [IQR]	14.1 [9.6–20.3]
Aspect ratio, median [IQR]	1.74 [0.97–2.76]
Volume (ml), median [IQR]	2.61 [1.58 to 3.96]
Surface area (mm²), median [IQR]	284.00 [201.9 to 386.7]
Wall thickness (mm), median [IQR]	1.81 [1.07 to 2.45]

median (interquartile range) when non-normally distributed, based on Shapiro–Wilk normality testing

*n* number of patients,* SD* Standard deviation; IQR, interquartile range

*indicates statistical significance (*p* < 0.05)

Median follow-up for the entire cohort was 53 months (IQR, 40.5–68). Patients with recurrence had a median follow-up of 54 months (IQR, 45.25–65.25), which was comparable to that of patients without recurrence (median 53 months; IQR, 40–69), indicating no meaningful difference in follow-up duration between groups. The overall recurrence rate at a minimum follow-up of 24 months was 8.6% (30/347). Dominant-hand involvement was significantly associated with recurrence (χ² *p* = 0.006) (Table 2) and remained a significant predictor in univariable logistic regression (OR = 5.19; 95% CI, 1.54–17.49; *p* = 0.007). Cyst distance to the adjacent joint was significantly shorter in recurrent cases compared with non-recurrent cases (mean 7.6 mm vs. 8.1 mm; t-test *p* = 0.001, Mann–Whitney U *p* = 0.021). When cyst-to-joint distance was analyzed categorically by quartiles, recurrence rates showed a graded decrease with increasing distance. Recurrence occurred in 12.2% of patients in the shortest-distance quartile compared with only 1.1% in the longest-distance quartile. The overall difference across quartiles was statistically significant (χ² = 8.46, *p* = 0.037) (Supplementary Table S1). In univariable logistic regression, shorter cyst-to-joint distance was associated with an increased risk of recurrence, with each additional millimeter conferring a relative risk reduction (OR = 0.66; 95% CI, 0.46–0.95; *p* = 0.028). In univariable analyses, age, sex, side of involvement, cyst location (dorsal vs. volar), cyst volume, surface area, wall thickness, longest and shortest diameters, and aspect ratio were not significantly associated with recurrence (all *p* > 0.05; Table 2).

**Table 2:** Factors associated with recurrence after open excision of wrist ganglion cysts

**Variable**	**No recurrence**	**Recurrence**	**Welch t-test *p***	**Mann–Whitney *p***	**Univariable OR (95% CI)**	***p* (logistic)**
Age (years), mean ± SD	42.2 ± 16.4	45.9 ± 15.4	0.210	0.252	1.014 (0.991–1.038)	0.229
Distance to joint (mm), mean ± SD	8.1 ± 1.2	7.6 ± 0.7	0.001*	0.021*	0.666 (0.463–0.959)	0.028*
Longest diameter (mm), median [IQR]	24.5 [14.6 to 39.3]	27.2 [12.3 to 40.0]	0.848	0.939	1.003 (0.975–1.031)	0.838
Shortest diameter (mm), median [IQR]	14.2 [8.4 to 22.5]	19.2 [10.3 to 25.6]	0.109	0.08	1.045 (0.996–1.096)	0.075
Aspect ratio, median [IQR]	1.74 [0.97 to 2.95]	1.62 [0.83 to 2.11]	0.057	0.282	0.839 (0.655–1.075)	0.164
Volume (ml), median [IQR]	2.61 [1.60 to 3.87]	3.00 [1.06 to 4.28]	0.709	0.603	1.067 (0.810–1.404)	0.646
Surface area (mm²), median [IQR]	284.50 [207.50 to 401.30]	240.55 [116.00 to 368.27]	0.111	0.099	0.741 (0.518–1.060)	0.100
Wall thickness (mm), median [IQR]	1.80 [1.07 to 2.45]	1.83 [1.12 to 2.26]	0.967	0.893	0.990 (0.603–1.624)	0.967
**Categorical variables**	**No recurrence**	**Recurrence**	**Test**	**p-value**	**Univariable OR (95% CI)**	**p (logistic)**
Dominant hand involved, n (%)	201 (63.4%)	27 (90.0%)	Chi-square	0.006*	5.194 (1.542–17.497)	0.007*
Female, n (%)	218 (68.8%)	22 (73.3%)	Chi-square	0.604	0.801 (0.345–1.861)	0.605
Dorsal location, n (%)	219 (69.1%)	16 (53.3%)	Chi-square	0.077	1.955 (0.918–4.164)	0.082
Side (Left vs. Right), n (%)	150 (47.3%)	17 (56.7%)	Chi-square	0.684	1.256 (0.593–2.661)	0.055

Continuous variables are presented as mean ± standard deviation when normally distributed and as median (interquartile range) when non-normally distributed, based on Shapiro–Wilk normality testing

*n* number of patients, *OR* odds ratio,* CI* Confidence interval, *SD* Standard deviation, *IQR* Interquartile range

*indicates statistical significance (*p* < 0.05)

The final exploratory multivariable model included three predictors for 30 recurrence events, corresponding to an EPV ratio of approximately 10. In the age-adjusted multivariable logistic regression, dominant-side involvement was associated with a significantly increased risk of recurrence (adjusted OR = 6.51; 95% CI, 1.87–22.63; *p* = 0.003). After adjustment for age, each additional millimeter of cyst-to-joint distance was associated with an approximately 41% relative reduction in recurrence risk (adjusted OR = 0.59; 95% CI, 0.39–0.90; *p* = 0.013). Sensitivity analyses adjusting separately for sex and cyst location yielded consistent results (Supplementary Table S1).

Interobserver agreement for MRI-based measurements was high across all parameters, with intraclass correlation coefficients ranging from 0.81 to 0.92. Specifically, the ICC for cyst-to-joint distance was 0.86 (95% CI, 0.82–0.90), supporting good reproducibility of distance measurements.

## Discussion

This study demonstrates that wrist GCs located on the dominant hand and those situated closer to the adjacent joint are more likely to recur after surgical excision. These findings suggest that selected clinical characteristics and MRI-based anatomical features may provide additional information when assessing recurrence risk.

Previous studies have explored recurrence risk primarily through demographic and procedural parameters. In the present cohort, neither age nor sex was associated with recurrence, consistent with several previous reports suggesting that demographic factors alone may not reliably predict postoperative recurrence of wrist GCs [11, 12]. Hand dominance, however, has been less frequently evaluated [10]. The increased recurrence risk in dominant hands observed here may reflect higher mechanical stress, repetitive microtrauma, and earlier functional demand after surgery, supporting closer surveillance and perhaps more protective postoperative protocols for these patients.

Imaging-based predictors have gained interest as MRI offers precise delineation of cyst morphology, stalk origin, and capsular relationships. Prior research has shown that incomplete resection of the cyst stalk or capsular connection is a leading cause of recurrence [13–16]. Our results confirm that cysts located nearer to the joint—likely with shorter stalks or a closer capsular connection—are at higher risk. We hypothesize that this finding may be attributable to incomplete surgical excision arising from difficulty in intraoperative delineation of the stalk, which tends to be shorter in cysts located close to the joint. The integration of measurements such as volume, wall thickness, and surface area into risk models remains limited; our study provides one of the more comprehensive MRI-based topographic assessments reported to date.

The recurrence rate of 8.6% in our cohort highlights that recurrence remains a concern even with surgical treatment, which is generally considered the most effective option compared with aspiration or observation [3, 8]. It should be noted that this recurrence rate reflects a minimum follow-up of 24 months, whereas several prior studies have reported recurrence outcomes at shorter follow-up intervals, which may partly account for differences in reported rates. This underscores the importance of refining risk assessment beyond general demographics and simple location categories.

Although the difference in cyst-to-joint distance between recurrent and non-recurrent cases was statistically significant, the absolute magnitude of this difference was relatively small (approximately 0.5 mm). This magnitude approaches the spatial resolution of routine wrist MRI and may overlap with measurement variability related to voxel size and slice selection. Accordingly, this parameter should not be interpreted as a surgically discernible or actionable threshold. Rather, we view cyst-to-joint distance as an imaging-derived anatomic risk marker reflecting closer capsular proximity, which may increase the technical difficulty of achieving complete stalk excision. Notably, the quartile-based analysis demonstrated a marked reduction in recurrence in the longest-distance quartile (1.1%), supporting a non-linear and threshold-independent association between distance and recurrence risk. Accordingly, cyst-to-joint distance should be regarded as a contributory anatomical factor that provides contextual insight into recurrence risk rather than a standalone clinical cutoff for surgical decision-making.

Because the exact joint of origin could not be consistently determined, cyst-to-joint distance should not be interpreted as a joint-specific parameter. Rather, it represents an imaging-derived marker of capsular proximity, which may increase the technical difficulty of complete stalk excision and thereby contribute to recurrence risk. In this context, the observed association likely reflects local anatomic constraints common to several wrist joints rather than pathology of a single joint.

This single-center cohort is relatively large, the surgical approach was uniform, and MRI-based topographic measurements were evaluated using multivariable analyses. However, several limitations should be acknowledged. The most important limitation of this study is the lack of standardized assessment of capsular and stalk management during surgery. As incomplete excision of the capsular origin is a well-established cause of recurrence, unmeasured variation in surgical technique (e.g., cautery versus curettage), surgeon experience, and postoperative immobilization protocols may have influenced recurrence risk and could not be adjusted for in the analysis. Therefore, the identified MRI-based associations should be interpreted as preoperative risk indicators rather than determinants of surgical success. The retrospective study design, potential variability in postoperative rehabilitation protocols, and the lack of external validation may limit the generalizability of the findings. Furthermore, several potentially important factors, such as precise lesion and stalk location, surrounding anatomical structures, symptom duration, symptom status, and MRI-derived stalk thickness, could not be reliably or consistently characterized in this retrospective dataset and were therefore not incorporated into the analysis. In addition, because this was a retrospective study based on an existing cohort, a priori sample size or power calculation was not performed. Future multicenter, prospective studies are warranted to confirm these risk factors, establish reliable MRI-derived cutoff values, and evaluate risk-adapted surgical strategies.

In summary, combining demographic variables such as hand dominance with MRI-derived cyst topography may improve risk assessment for postoperative recurrence. Incorporating these findings into preoperative planning may help surgeons tailor operative technique, set realistic patient expectations, and potentially reduce recurrence rates.

## Conclusions

Dominant-hand involvement and shorter distance between the cyst and adjacent joint may be independently associated with recurrence after ganglion cyst excision. These parameters can be considered when counseling patients and planning surgical strategies to minimize recurrence.

## Supplementary Information

Supplementary Material 1: Quartile-based recurrence rates and sensitivity analyses.

## Data Availability

The datasets used and analysed during the current study are available from the corresponding author on reasonable request.

## Abbreviations

GCs: Ganglion cysts
MRI: Magnetic resonance imaging
OR: Odds ratio
CI: Confidence interval
PACS: Picture archiving and communication system
DICOM: Digital imaging and communications in medicine
IRB: Institutional review board
IQR: Interquartile range
EPV: Events-per-variable
ICCs: Intraclass correlation coefficients
