# Supplementary material for: Predictors of recurrence after open excision of wrist ganglion cysts: an MRI-based and clinical analysis
Source: BMC Surg. 2026 Feb 5;26:182. doi: 10.1186/s12893-026-03562-3 (PMC12964805; doi:10.1186/s12893-026-03562-3)
Supplement: Supplementary file 1 — Supplementary Material 1: Quartile-based recurrence rates and sensitivity analyses. [file 12893_2026_3562_MOESM1_ESM.docx]

| **Cyst-to-joint distance quartile** | **Total (n)** | **No recurrence, n (%)** | **Recurrence, n (%)** |
| --- | --- | --- | --- |
| Q1 (shortest distance) | 90 | 79 (87.8) | 11 (12.2) |
| Q2 | 84 | 75 (89.3) | 9 (10.7) |
| Q3 | 86 | 77 (89.5) | 9 (10.5) |
| Q4 (longest distance) | 87 | 86 (98.9) | 1 (1.1) |
| Overall | 347 | 317 (91.4) | 30 (8.6) |

Panel A. Recurrence according to cyst-to-joint distance quartiles

Statistical test: Chi-square test for trend (or Pearson χ²) *p* = 0.037. Percentages represent recurrence rates within each quartile.

Panel B. Sensitivity analyses (multivariable logistic regression)

| **Model** | **Variable** | **OR** | **95% CI** | **p** |
| --- | --- | --- | --- | --- |
| Unadjusted | Dominant hand | 6.43 | 1.86–22.25 | 0.003* |
|  | Distance to joint (mm) | 0.58 | 0.38–0.88 | 0.010* |
| Age-adjusted (primary) | Dominant hand | 6.51 | 1.87–22.63 | 0.003* |
|  | Distance to joint (mm) | 0.59 | 0.39–0.90 | 0.013* |
|  | Age | 1.01 | 0.99–1.04 | 0.350 |
| Sex-adjusted | Dominant hand | 6.39 | 1.85–22.13 | 0.003* |
|  | Distance to joint (mm) | 0.58 | 0.39–0.88 | 0.011* |
|  | Sex | 0.86 | 0.36–2.05 | 0.740 |
| Location-adjusted | Dominant hand | 6.19 | 1.79–21.43 | 0.004* |
|  | Distance to joint (mm) | 0.61 | 0.40–0.92 | 0.018* |
|  | Dorsal location | 0.62 | 0.28–1.36 | 0.240 |

OR, odds ratio; CI, confidence interval; * indicates statistical significance (*p* < 0.05).
